# Laugh and Grow Fat

**Published:** 1904-12

**Authors:** 


### "LAUGH AND GROW FAT."

Life is such a serious business with the average mortal that an opportunity for a hearty laugh is more than welcome to most people. "A merry heart doeth good like a medicine," and so do the humorous features of that great metropolitan daily, *The Chicago-Record Herald*. The first thing that greets you on the first page at every issue is the humorous cartoon by Ralph Wilder, the well-known artist, that frequently tells more at a glance than could be conceived in a column of reading matter. Every issue contains also a humorous small story on the editorial page, and the "Alternating Currents" column, written by S. E. Kiser, one of the most popular humorous writers in the country. In addition to all these, the Sunday issue always includes a comic section, guaranteed to produce laughter.

